# Fixation Probabilities for Any Configuration of Two Strategies on Regular Graphs

**DOI:** 10.1038/srep39181

**Published:** 2016-12-22

**Authors:** Yu-Ting Chen, Alex McAvoy, Martin A. Nowak

**Affiliations:** 1 Program for Evolutionary Dynamics, Harvard University, Cambridge, MA 02138, USA; 2 Center of Mathematical Sciences and Applications, Harvard University, Cambridge, MA 02138, USA; 3 Department of Mathematics, University of Tennessee, Knoxville, TN 37996, USA; 4 Department of Mathematics, University of British Columbia, 1984 Mathematics Road, Vancouver, BC, Canada V6T 1Z2; 5 Department of Mathematics, Harvard University, Cambridge, MA 02138, USA; 6 Department of Organismic and Evolutionary Biology, Harvard University, Cambridge, MA 02138, USA.

## Abstract

Population structure and spatial heterogeneity are integral components of evolutionary dynamics, in general, and of evolution of cooperation, in particular. Structure can promote the emergence of cooperation in some populations and suppress it in others. Here, we provide results for weak selection to favor cooperation on regular graphs for any configuration, meaning any arrangement of cooperators and defectors. Our results extend previous work on fixation probabilities of rare mutants. We find that for any configuration cooperation is never favored for birth-death (BD) updating. In contrast, for death-birth (DB) updating, we derive a simple, computationally tractable formula for weak selection to favor cooperation when starting from any configuration containing any number of cooperators. This formula elucidates two important features: (i) the takeover of cooperation can be enhanced by the strategic placement of cooperators and (ii) adding more cooperators to a configuration can sometimes suppress the evolution of cooperation. These findings give a formal account for how selection acts on all transient states that appear in evolutionary trajectories. They also inform the strategic design of initial states in social networks to maximally promote cooperation. We also derive general results that characterize the interaction of any two strategies, not only cooperation and defection.

Mechanisms favoring the emergence of cooperation in social dilemmas have become central focuses of evolutionary game theory in recent years^1^,^2^,^3^. The dilemma of cooperation, which is characterized by conflicts of interest between individuals and groups, poses a significant challenge to models of evolution since many of these models predict that cooperation cannot persist in the presence of exploitation by defectors^4^,^5^. Yet cooperation is widely observed in nature, and the spatial assortment that results from population structure is one element that can promote its emergence. In fact, spatial structure is among the most salient determinants of the evolutionary dynamics of a population^6^,^7^,^8^,^9^,^10^,^11^,^12^,^13^,^14^,^15^,^16^,^17^,^18^,^19^,^20^,^21^,^22^,^23^,^24^,^25^,^26^,^27^,^28^.

In social dilemmas, population structure can allow for the emergence of localized cooperative clusters that would normally be outcompeted by defectors in well-mixed populations^5^,^29^. However, whether population structure promotes or suppresses cooperation depends on a number of factors such as the update rule, the type of social dilemma, and the spatial details of the structure (which determine the extent of local competition see refs 28 and 30). For example, cooperation need not be favored in prisoner’s dilemma interactions under all update rules^31^,^32^,^33^,^34^. As a consequence, population structure should be considered in the context of the game and the underlying update rule.

In the donation game, a cooperator (*C*) pays a cost, *c*, to provide the opponent with a benefit, *b*, and a defector (*D*) pays no cost and provides no benefit^35^. Provided *b* > *c* > 0, this game represents a prisoner’s dilemma since then the unique Nash equilibrium is mutual defection, but both players would prefer the payoff from mutual cooperation^36^. In addition to representing one of the most important social dilemmas, the donation game also admits a simple way in which to quantify the efficiency of cooperation: the benefit-to-cost ratio, *b*/*c*. As this ratio gets larger, the act of cooperation has a more profound effect on the opponent relative to the cost paid by the cooperator. As we shall see, for any configuration of cooperators and defectors, this ratio is a vital indicator of the evolutionary performance of cooperation.

Evolutionary graph theory is a framework for studying evolution in structured populations^30^,^32^,^33^,^37^,^38^,^39^,^40^,^41^,^42^,^43^,^44^,^45^,^46^,^47^. In a graph-structured population, the players reside on the vertices and the edges indicate who is a neighbor of whom. In fact, there are two types of neighborhoods: (i) those that generate payoffs (“interaction neighborhoods”) and (ii) those that are relevant for evolutionary updating (“dispersal neighborhoods”). Thus, an evolutionary graph is actually a pair of graphs consisting of an interaction graph and a dispersal graph^46^,^48^,^49^,^50^. As in many other studies, we assume that the interaction and dispersal graphs are the same. Other extensions of evolutionary graph theory involve dynamic graphs, which allow the population structure to change during evolutionary updating^51^,^52^,^53^,^54^. Our focus is on static, regular graphs of degree *k*, meaning the population size, *N*, is fixed and each player has exactly *k* neighbors.

We study two prominent update rules: birth-death (BD) and death-birth (DB). In both processes, players are arranged on a graph and accumulate payoffs by interacting with all of their neighbors. This payoff, *π*, is then converted to fitness, *f*, via *f* = 1 + *wπ*, where *w* ≥ 0 is the intensity of selection^5^. For BD updating^5^,^55^, a player is chosen with probability proportional to fitness for reproduction; the offspring of this player then replaces a random neighbor (who dies). For DB updating^32^, a player is chosen uniformly at random for death; a neighbor of this player then reproduces (with probability proportional to fitness) and the offspring fills the vacancy. For each of these processes, we assume that *w* is small, which means selection is weak. Weak selection is often a biologically meaningful assumption since an individual might possess many traits (strategies), and each trait makes only a small contribution to fitness^5^,^56^,^57^,^58^,^59^,^60^,^61^.

The effects of selection on fixation probability have been studied chiefly for states with just a single cooperator since, if the mutation rate is small, the process will reach a monomorphic state prior to the appearance of another cooperator through mutation^62^,^63^. Although small mutation rates are often reasonable from a biological standpoint^64^,^65^,^66^,^67^, there are several reasons to study arbitrary cooperator configurations. Even when starting from a state with a single cooperator, an evolutionary process typically transitions subsequently through states with many cooperators. From a mathematical standpoint, it is therefore natural to ask how selection affects the fixation probability of cooperators from each possible transient state that might arise in an evolutionary trajectory. Furthermore, many-mutant states could arise through migration^68^,^69^,^70^,^71^ or environmental mutagenic agents^72^,^73^, which, even when rare, might result in several cooperators entering the population at once. In the case of social networks, cooperators could arise through design rather than mutation or exploration; if cooperators can be strategically planted within the population, then one can ask how to do so in order to maximize the chances that cooperators take over. Therefore, the effects of selection on arbitrary numbers and configurations of cooperators and defectors play an important role in the evolutionary dynamics of cooperation.

When starting from a configuration with *n* cooperators and *N* − *n* defectors, weak selection is said to favor the evolution of cooperation (on a regular graph) if the probability that cooperators fixate exceeds *n*/*N* if *w* is sufficiently small but positive. This comparison is based on the fact that the fixation probability of *n* cooperators for neutral drift (*w* = 0) is *n*/*N*. Ohtsuki *et al*.^32^ show that, on large regular graphs of degree *k*, selection favors the fixation of a single, randomly-placed cooperator under DB updating as long as


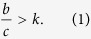


Taylor *et al*.^46^ show that for finite bi-transitive graphs of size *N* and degree *k*, the condition for selection to favor the fixation of a single cooperator is


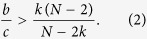


Bi-transitive graphs constitute a subset of regular graphs.

In another refinement of the ‘*b*/*c* > *k*’ result, Chen^39^ shows that, for any *n* with 0 < *n* < *N*, selection favors cooperation when starting from a random configuration of *n* cooperators and *N* − *n* defectors on a regular graph of size *N* and degree *k* if and only if Eq. (2) holds. This ratio, which characterizes when selection increases the fixation probability of cooperators, is independent of the location of the mutants, despite the fact that the probability of fixation itself depends on the location^74^. As the population size, *N*, gets large, the critical benefit-to-cost ratio of Eq. (2) approaches *k*, which recovers the result of Ohtsuki *et al*.^32^. Our goal here is to move beyond Eq. (2) and give an explicit, computationally feasible critical benefit-to-cost ratio for any configuration of cooperators and defectors on any regular graph.

Given the profusion of possible ways to structure a population of a fixed size, it quickly becomes difficult to determine when a population structure favors the evolution of cooperation. Here, we provide a solution to this problem for BD and DB updating on regular graphs. We show that, for any configuration of cooperators and defectors, (i) cooperation is never favored for BD updating, and (ii) for DB updating, there exists a simple, explicit critical benefit-to-cost ratio that characterizes when selection favors the emergence of cooperation. Moreover, if *N* is the population size and *k* is the degree of the graph, then the complexity of calculating this ratio is *O*(*k*^2^*N*), and, in particular, linear in *N*. Thus, while the calculations of fixation probabilities in structured populations are famously intractable^75^,^76^,^77^, the determination of whether or not selection increases the probability of fixation, for weak selection, is markedly simpler.

In addition to providing a computationally feasible way of determining whether selection favors cooperation on a particular graph, our results highlight the importance of the initial configuration for the emergence of cooperation. Depending on the graph, adding additional cooperators to the initial condition can either suppress or promote the evolution of cooperation. A careful choice of configuration of cooperators and defectors can minimize the critical benefit-to-cost ratio for selection to favor cooperation. If cooperation is not favored by selection in such a strategically chosen initial state, then it cannot be favored under any other initial configuration. In this sense, there exists a configuration that is most conducive to the evolution of cooperation, which is not apparent from looking at single-cooperator configurations or random configurations with *n* cooperators since these initial configurations need not minimize the critical benefit-to-cost ratio.

## Results

### Critical benefit-to-cost ratios

Let *ξ* be a configuration of cooperators and defectors on a fixed regular graph of size *N* and degree *k*, and let **C** denote the configuration consisting solely of cooperators. For the donation game, the probability that cooperators take over the population when starting from state *ξ* may be viewed as a function of the selection intensity, *ρ*_*ξ*,**C**_(*w*). We consider here the following question: when does weak selection increase the probability that cooperators fixate? In other words, when is *ρ*_*ξ*,**C**_(*w*) > *ρ*_*ξ*,**C**_(0) for sufficiently small *w* > 0? Note that if there are *n* cooperators in state *ξ*, then *ρ*_*ξ*,**C**_(0) = *n*/*N*, so this condition is equivalent to *ρ*_*ξ*,**C**_(*w*) > *n*/*N* for small *w* > 0.

**Figure 1:**
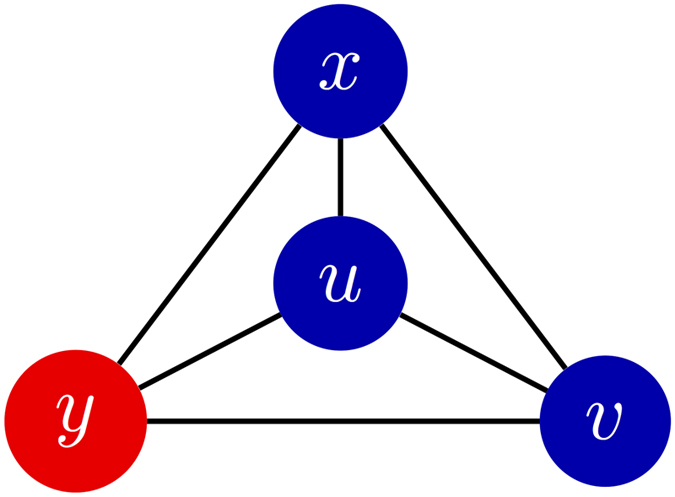
Calculation of the local frequencies of Eqs (3),(4),(5),(6), *f*_1_(*x, ξ*), *f*_0_(*x, ξ*), and *f*_10_(*x, ξ*), where *ξ* is the configuration consisting of a defector at vertex *y* and cooperators elsewhere. Among the three neighbors of the player at vertex *x*, two are cooperators (*u* and *v*) and one is a defector (*y*); thus, *f*_1_(*x, ξ*) = 2/3 and *f*_0_(*x, ξ*) = 1/3. Furthermore, of the nine paths of length two that begin at vertex *x*, only two (*x* → *u* → *y* and *x* → *v* → *y*) consist of a cooperator followed by a defector, and it follows that *f*_10_(*x, ξ*) = 2/9.

To answer this question, we first need to introduce some notation. If *x* is a vertex of the graph and *ξ* is a configuration, then let *f*_1_(*x, ξ*) and *f*_0_(*x, ξ*) be the frequencies of cooperators and defectors, respectively, among the neighbors of the player at vertex *x*. Similarly, let *f*_10_(*x, ξ*) be the fraction of paths of length two, starting at *x*, that consist of a cooperator followed by a defector. From these quantities, let


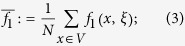



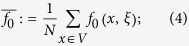



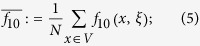



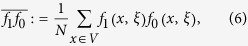


which are obtained by averaging these ‘local frequencies’ over all of the players in the population. From these local frequencies, which are straightforward to calculate (see Fig. 1), we obtain our main result: for small *w* > 0, *ρ*_*ξ*,**C**_(*w*) > *ρ*_*ξ*,**C**_(0) if and only if the benefit-to-cost ratio exceeds the critical value


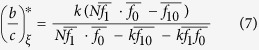


whenever the denominator is positive (and ∞ otherwise). Since the calculations of 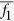, 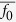, and 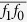 are *O*(*kN*) and the calculation of 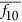 is *O*(*k*^2^*N*), it follows that the complexity of finding the critical benefit-to-cost ratio is *O*(*k*^2^*N*), so it is feasible to calculate even when the population is large. Note also that if 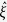 is the state obtained by swapping cooperators and defectors in *ξ*, then both *ξ* and 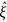 have the same critical benefit-to-cost ratio. We discuss these ‘conjugate’ states further in our treatment of structure coefficients.

When *ξ* has just a single cooperator, the ratio of Eq. (7) reduces to that of Eq. (2), which, in particular, does not depend on the location of the cooperator. This property is notable because the fixation probability itself usually does depend on the location of the cooperator, even on regular graphs^74^. We show in Methods that one recovers from Eq. (7) the result of Chen^39^ that Eq. (2) gives the critical benefit-to-cost ratio for a randomly-chosen configuration with a fixed number of cooperators.

For fixed *k* ≥ 2, the critical benefit-to-cost ratio in Eq. (7) converges uniformly to *k* as *N* → ∞ (see Methods). Therefore, on sufficiently large graphs, the critical ratio is approximated by *k* for any configuration, regardless of the number of cooperators. As a result, on large graphs there is less of a distinction between the various transient (non-monomorphic) states in terms of whether or not selection favors the fixation of cooperators. On smaller graphs, these transient states can behave quite differently from one another. This effect is particularly pronounced on very small social networks in which cooperators can be strategically planted in the population to ensure that cooperators are favored by selection.

#### Strategic placement of cooperators in (small) social networks

Among the more interesting consequences of Eq. (7) are its implications for the success of cooperators as a function of the initial configuration. Recall that Eq. (2) gives the critical benefit-to-cost ratio for both (i) configurations with a single cooperator and (ii) random configurations with a fixed number of cooperators. When cooperators and defectors are configured randomly, this critical ratio is independent of the number of cooperators, which suggests that the effects of selection cannot be improved by increasing the initial abundance of cooperators.

**Figure 2:**
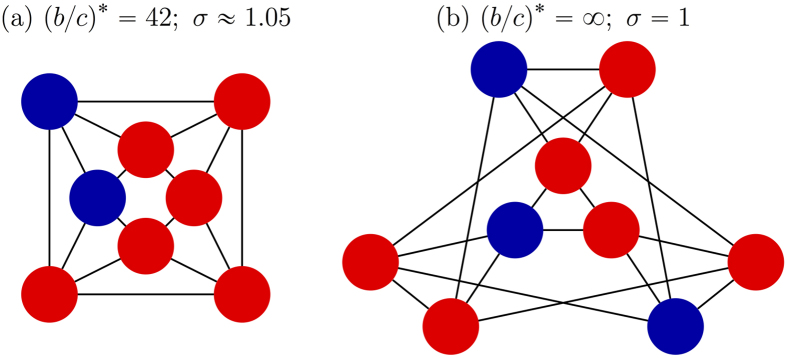
Two graphs showing configurations of cooperators (blue) and defectors (red). (**a**) Cooperation can be favored for the initial condition that is shown since the critical benefit-to-cost ratio is 42 and, in particular, finite. However, the fixation of cooperation cannot be favored for any initial configuration with a single cooperator on this graph. (**b**) Cooperation cannot be favored for the initial condition that is shown since the critical benefit-to-cost ratio is infinite. However, any initial configuration with a single cooperator has a critical benefit-to-cost ratio of 28. Therefore, the addition of cooperators to the initial configuration can either favor cooperation, (**a**), or suppress it, (**b**). The critical benefit-to-cost ratio can also be expressed in terms of a well-known quantity known as a “structure coefficient”, *σ*, which satisfies (*b*/*c*)^*^ = (*σ* + 1)/(*σ* − 1).

Equation (7), on the other hand, shows that the initial configuration of cooperators, including their abundance, does affect how selection acts on the population. First of all, there are graphs for which the critical benefit-to-cost ratio is infinite for configurations with a single cooperator but finite for some configurations with multiple cooperators (see Fig. 2(a)). In contrast, there are graphs for which this ratio is finite for configurations with a single cooperator but infinite for some states with multiple cooperators (see Fig. 2(b)). Therefore, despite the fact that the critical ratio for a single mutant is the same as the critical ratio for a random configuration with any fixed number of mutants, the critical ratio does (in general) depend on the number of mutants present in the configuration.

**Figure 3:**
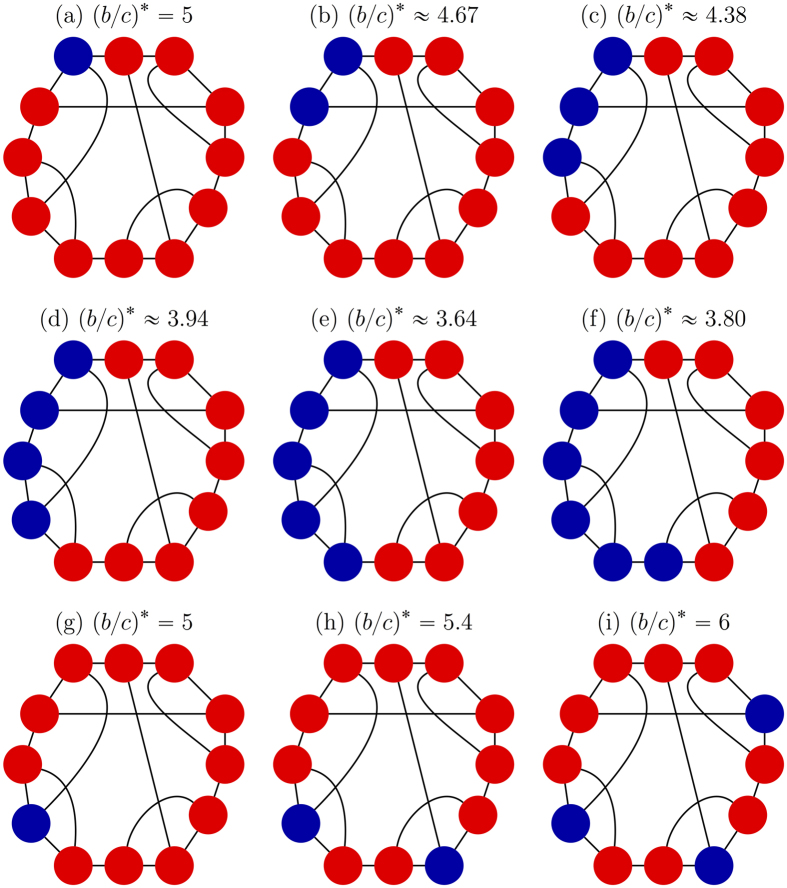
Configurations of cooperators and defectors on the Frucht graph, a 3-regular graph with 12 vertices and no non-trivial symmetries; see ref. 85. Panels (a–f) show the effects on the critical benefit-to-cost ratio of adding additional cooperators to the initial state. Panel (e) shows the global minimum of 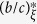, which is achieved by just (**e**) and its conjugate; adding additional cooperators to the configuration in (**e**) only increases 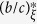. The configuration of (e) is ‘optimal’ for cooperation in the sense that if selection increases the fixation probability of cooperators in some state, then it does so in state (**e**) as well. Relative to all possible initial states, selection can increase the fixation probability of cooperators in (**e**) under the smallest *b*/*c* ratio. Panels (g–i) show that when cooperators are added in a different order (starting with just a single cooperator), the critical benefit-to-cost ratio can actually be increased. Each of these three configurations has isolated cooperators, and (**i**) gives the global maximum of 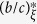, which is achieved by just (**i**) and its conjugate. Since *N*_0_ = 3, (**i**) is a maximal isolated configuration. The initial state in (**i**) is least conducive to cooperation in the sense that, relative to all other initial configurations, (**i**) requires the largest *b*/*c* ratio for selection to increase the fixation probability of cooperators. If selection increases this fixation probability when starting from state (**i**), then it does so when starting from any other mixed initial configuration.

We say that a configuration has isolated cooperators (resp. defectors) if the minimum distance between any two cooperators (resp. defectors) is at least three steps. Let *N*_0_ denote the maximum number of isolated strategies that a configuration can carry. (Examples of configurations with isolated cooperators on a graph with *N*_0_ = 3 are given in Fig. 3). If a strategy (cooperate or defect) appears only once in a configuration, then that strategy is clearly isolated, so *N*_0_ ≥ 1. We show in Methods that if *N* > 2*k*, then cooperation can be favored for a mixed initial condition with *n* cooperators whenever 1 ≤ *n* ≤ *N*_0_ + 1 or 1 ≤ *N* − *n* ≤ *N*_0_ + 1, and, moreover, these bounds on *n* are sharp. Stated differently, under these conditions any configuration with *n* cooperators has a finite critical benefit-to-cost ratio. Furthermore, if *N*_0_ ≥ 2, then, for any *n* with 2 ≤ *n* ≤ *N*_0_, there exists a configuration with *n* cooperators whose critical ratio is strictly less than the ratio for a single cooperator (Eq. (2)). Such a configuration necessarily has no isolated strategies since the minimum critical ratio among configurations with an isolated strategy is attained by any state with just a single cooperator.

The strategic placement of cooperators and defectors can therefore produce a critical benefit-to-cost ratio that is less than the ratio for a single cooperator among defectors. In fact, starting from a configuration with just one cooperator, one can reduce this critical ratio by placing a second cooperator adjacent to the first cooperator (see Methods). If *b*/*c* lies below Eq. (2) and above Eq. (7), then a strategically chosen configuration can ensure that the fixation of cooperation is favored by selection even if it is disfavored for any single-cooperator state. This behavior is particularly pronounced on small social networks, where the critical ratios take on a significant range of values (see Fig. 3), and less apparent on large networks, where the critical ratios are much closer to the degree of the graph, *k*. Fortunately, on small networks it is easier to directly search for configurations that have small critical benefit-to-cost ratios via Eq. (7).

### Structure coefficients

Consider now a generic 2 × 2 game whose payoff matrix is


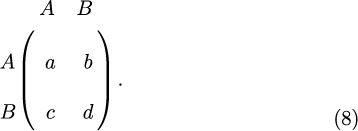


The donation game is a special case of this game with *A* indicating a cooperator and *B* indicating a defector. If **A** denotes the monomorphic state consisting of only *A*-players and if *ξ* is a configuration of *A*- and *B*-players, then a natural generalization of the question we asked for the donation game is the following: when is *ρ*_*ξ*,**A**_(*w*) > *ρ*_*ξ*,**A**_(0) for sufficiently small *w* > 0? That is, when does (weak) selection favor the fixation of *A* when starting from state *ξ*? For technical reasons, this question is more difficult to answer when the payoff matrix is Eq. (8) instead of that of the donation game. There is, however, an alternative way of generalizing the critical benefit-to-cost ratio to Eq. (8).

When considering the evolutionary success of strategy *A* based on configurations with only one mutant, another standard measure is whether the fixation probability of a single *A*-mutant in a *B*-population exceeds that of a single *B*-mutant in an *A*-population [see ref. 34, Eq. 2]. That is, one compares the fixation probability of *A* to the fixation probability of *B* after swapping *A* and *B* in the initial state. This interchange of strategies may be defined for any initial state: formally, if *ξ* is a configuration of *A*-players and *B*-players, the conjugate of *ξ*, written 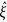, is the state obtained by swapping *A* and *B* in *ξ*. In other words, the *A*-players in *ξ* are the *B*-players in 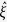.

A natural generalization of this criterion to arbitrary configurations involves comparing the fixation probability of *A* in *ξ* to the fixation probability of *B* in 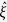. Let **A** and **B** be the monomorphic states consisting of all *A*-players and all *B*-players, respectively. In this context, our main result is that 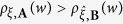 for all sufficiently small *w* > 0 if and only if


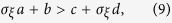


where, for DB updating,


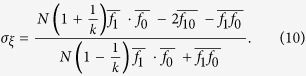


In Methods, we give an explicit formula for the structure coefficient, *σ*_*ξ*_, for BD updating as well. Just as it is for the critical benefit-to-cost ratio of Eq. (7), the complexity of calculating *σ*_*ξ*_ is *O*(*k*^2^*N*). In fact, the relationship between 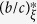 and *σ*_*ξ*_ is remarkably straightforward:


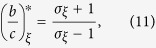


which, for DB updating, generalizes a result of Tarnita *et al*.^34^ to arbitrary configurations. Note that the critical benefit-to-cost ratio increases as *σ*_*ξ*_ decreases. Moreover, unlike the critical benefit-to-cost ratio, *σ*_*ξ*_ is always finite. Interestingly, both 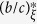 and *σ*_*ξ*_ are invariant under conjugation, meaning they are the same for 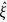 as they are for *ξ*.

For the donation game, Eq. (9) is equivalent to 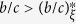. Of course, Eq. (9) applies to a broader class of games as well and represents a simple way to compare the success of a strategy (*A*) relative to its alternative (*B*) when selection is weak. In this sense, Eq. (9) may be thought of as a generalization of the critical benefit-to-cost rule to arbitrary 2 × 2 games.

## Discussion

Selection always opposes the emergence of cooperation for BD updating, regardless of the configuration of cooperators and defectors (see Methods). This result is consistent with previous studies showing that cooperation cannot be favored under random configurations^32^,^33^,^78^,^79^, and it specifies further that cooperation cannot be favored under any configuration. For general 2 × 2 games given by Eq. (8), we show in Methods that one can also find a simple formula for *σ*_*ξ*_ in the selection condition of Eq. (9) that can be easily calculated for a given graph.

Remarkably, for DB updating, both the critical benefit-to-cost ratio and *σ*_*ξ*_ depend on only local properties of the configuration, which makes these quantities straightforward to calculate. Furthermore, the complexity of calculating both of these quantities is *O*(*k*^2^*N*), where *N* is the size of the population and *k* is the degree of the graph, so they are computationally feasible even on large graphs. Therefore, our results provide a tractable way of determining whether or not selection favors cooperation for any configuration.

Finding an optimal configuration, which is one that minimizes the critical benefit-to-cost ratio, seems to be a difficult nonlinear optimization problem. The critical ratio is easily computed for any given configuration, but a graph of size *N* has 2^*N*^ possible configurations, which makes a brute-force search unfeasible for all but small *N*. Our results qualitatively show that both the abundance and the configuration of cooperators can strongly influence the effects of selection. We leave as an open problem whether it is possible to find a polynomial-time algorithm that produces an optimal configuration on any regular graph. However, since Eq. (7) is extremely easy to compute for a given configuration, and since small graphs generally exhibit broader variations of critical ratios than do larger graphs (since 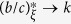 uniformly as *N* → ∞), it is typically feasible to find a state that is more conducive to cooperation than a random configuration.

Our analysis of arbitrary configurations uncovers two important features of the process with DB updating: (i) there exist graphs that suppress the spread of cooperation when starting from a single mutant but promote the spread of cooperation when starting from configurations with multiple mutants (Fig. 2(a)), and (ii) there exist graphs that promote the spread of cooperation when starting from a single mutant but suppress the spread of cooperation when starting from configurations with many mutants (Fig. 2(b)). The proper initial configuration is thus a crucial determinant of the evolutionary dynamics, and our results help to engineer initial conditions that promote the emergence of cooperation on social networks. More importantly, these results provide deeper mathematical insights into the complicated problem of how selection affects the outcome of an evolutionary process at each point along an evolutionary trajectory.

## Methods

### Notation and general setup

In what follows, the population structure is given by a simple, connected, *k*-regular graph, *G* = (*V, E*), where *V* denotes the vertex set of *G* and *E* denotes the edge set. For *x, y* ∈ *V*, we write *x*~*y* to indicate that *x* and *y* are neighbors, i.e. (*x, y*) ∈ *E*. Throughout the paper, we assume that #*V* = *N* is finite and *k* ≥ 2.

The payoff matrix for a generic game with strategies *A* and *B* is


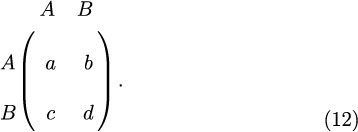


A configuration on *G*, denoted *ξ*, is a function from *V* to {0, 1}. If *ξ*(*x*) = 1, then the player at vertex *x* is using *A*; otherwise, this player is using *B*. A special case of Eq. (12) is the donation game,


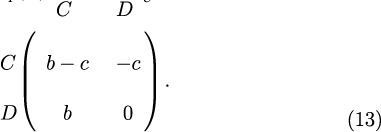


When we are considering the donation game, *ξ*(*x*) = 1 indicates a cooperator at vertex *x* and *ξ*(*x*) = 0 indicates a defector at vertex *x*. For any such configuration, *ξ*, the conjugate configuration, 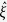, is defined as 
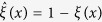
 for *x* ∈ *V*. In other words, 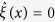 if *ξ*(*x*) = 1 and 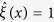 if *ξ*(*x*) = 0.

For any configuration, *ξ*, on a *k*-regular graph, *G*, and for *x* ∈ *V* and *i, j* ∈ {0, 1}, let


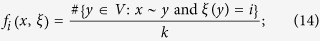



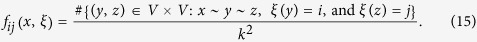


For any function, *f*(*x, ξ*), let


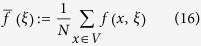


be the arithmetic average of *f* with respect to the vertices of *G*. (Fig. 1 in the main text gives an example of how these quantities are calculated). The arithmetic averages of the functions formed from these local frequencies admit simple probabilistic interpretations: If a random walk is performed on the graph at a starting point chosen uniformly-at-random, then 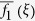 (resp. 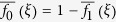) is the probability that the player at the first step is a cooperator (resp. a defector), and 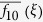 is the probability that the player at the first step is a cooperator and the player at the second step is a defector. If two independent random walks are performed at the same starting point, then 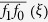 is the probability of finding a cooperator at step one in the first random walk and a defector at step one in the second random walk. If one chooses an enumeration of the vertices and represents *G* by an adjacency matrix, Γ, and *ξ* as a column vector, then


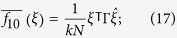



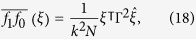


which gives a simple, alternative way to calculate each of 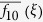 and 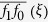.

Let *w* ≥ 0 be a sufficiently small selection intensity. The effective payoff of an *i*-player at vertex *x* in configuration *ξ*, denoted 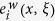, for the game whose payoffs are given by the generic matrix of Eq. (12), is defined via


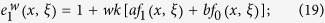



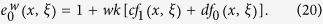


The basic measure we use here to define the evolutionary success of a strategy is fixation probability. If *X* is a strategy (either in {*A, B*} or in {*C, D*}), let **X** denote the monomorphic configuration in which every player uses *X*. For any configuration, *ξ*, and a fixed game, we write *ρ*_*ξ*,**X**_(*w*) to denote the probability that strategy *X* fixates in the population given an initial configuration, *ξ*, and selection intensity, *w*.

In the following sections, we consider DB and BD updating under weak selection (*w* ≪ 1).

### DB updating

Under DB updating, a player is first selected for death uniformly-at-random from the population. The neighbors of this player then compete to reproduce, with probability proportional to fitness (effective payoff), and the offspring of the reproducing player fills the vacancy. We assume that the strategy of the offspring is inherited from the parent. Therefore, if the player at vertex *x* dies when the state of the population is *ξ*, then the probability that this vacancy is filled by an *i*-player is


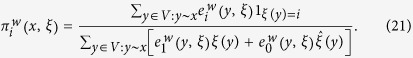


This DB update rule defines a rate-*N* pure-jump Markov chain, where *N* is the size of the population. When *w* = 0, this process reduces to the voter model such that, at each update time, a random individual adopts the strategy of a random neighbor; see ref. 80.

#### Critical benefit-to-cost ratios

Recall that our goal is to determine when, for any configuration, *ξ, ρ*_*ξ*,**C**_(*w*) > *ρ*_*ξ*,**C**_(0) for all sufficiently small *w* > 0. We first need some technical lemmas:

**Lemma 1.** For any configuration, *ξ*, we have the following first-order expansion as *w* → 0^+^:


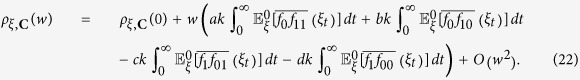


*Proof*. By Theorem 3.8 in ref. 39, we have


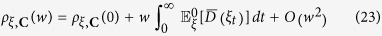


whenever *w* is sufficiently small, where


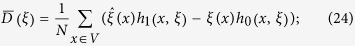



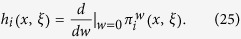


By the definition of 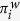, Eq. (21), we have


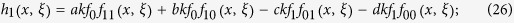



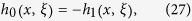


so Eq. (22) follows at once from Eq. (23), which completes the proof.□

**Remark 1.** The approach of studying fixation probabilities via first-order expansions, as in Eq. (23), also appears in refs 81, 82, 83. The proof of Eq. (23) in ref. 39, which is valid under mild assumptions on the game dynamics, was obtained independently and is a particular consequence of a series-like expansion for fixation probabilities. In addition to the identification of the first-order coefficients 
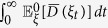
 in selection strength, *w*, in Eq. (23), the proof of this series-like expansion obtains a bound for the *O*(*w*^2^) error terms that is explicit in selection strength and the rate to reach monomorphic configurations of the underlying game dynamics. Therefore, one can deduce an explicit range of selection strengths such that the comparison of fixation probabilities requires only the sign of 
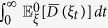
. We refer the reader to ref. 60 for a further discussion of selection strengths and their consequences for the comparison of fixation probabilities.

In order to compute the voter-model integrals in Eq. (22), we now turn to coalescing random walks on graphs. Suppose that {*B*^*x*^}_*x*__∈__*V*_ is a system of rate-1 coalescing random walks on *G*, where, for each *x* ∈ *V, B*^*x*^ starts at *x*. These interacting random walks move independently of one another until they meet, and thereafter they move together. The duality between the voter model and these random walks is given by


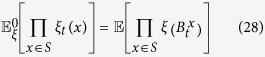


for each *S* ⊆ *V, t* > 0, and strategy configuration, *ξ*. For more information on this duality, including a proof of Eq. (28) and its graphical representation, see §III.4 and §III.6 in ref. 80.

Consider now two discrete-time random walks on *G*, 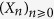 and 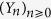, that start at the same vertex and are independent of {*B*^*x*^}_*x*∈*V*_. If the common starting point is *x* ∈ *V*, then we write 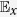 to denote the expectation with respect to this starting point. If the starting point is chosen with respect to the uniform distribution, *π*, then this expectation is denoted by 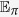. The random-walk probabilities, 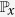 and 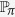, are understood in the same way. Since 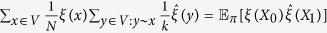, for example, we will use these random walks to save notation when we compute the local frequencies of strategy configurations.

**Lemma 2.** If f_*0_ := *f*_10_ + *f*_00_, then, for any configuration, *ξ*, we have


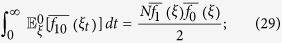



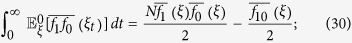



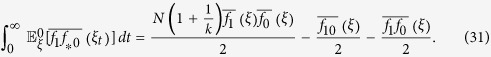


*Proof*. For any configuration, *ξ*, and any *t* > 0,


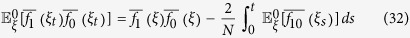


by Theorem 3.1 in ref. 84. See also Section 3 in that reference for discussions and related results of Eq. (32) in terms of coalescing random walks. Moreover, for any vertices *x* and *y* with *x* ≠ *y*, we have


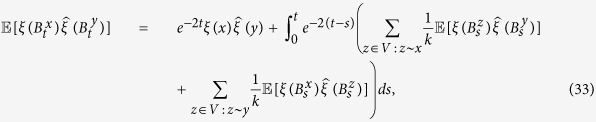


which is obtained by considering whether the first epoch time of the bivariate Markov chain (*B*^*x*^, *B*^*y*^) occurs before time *t* or not. Notice that Eq. (33) is false if *x* = *y* since the left-hand side vanishes but the integral term on the right-hand side is, in general, nonzero. This fact needs to be kept in mind when Eq. (33) is applied. Furthermore, using the duality of Eq. (28), the voter-model integrals in question are


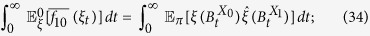



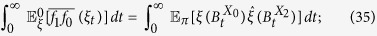



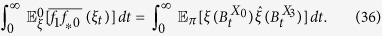


We are now in a position to establish Eqs (29),(30),(31). By letting *t* → ∞ in Eq. (32), we obtain Eq. (29) since 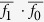 vanishes at monomorphic configurations. Since the graph has no self-loops, we have *X*_0_ ≠ *X*_1_ almost surely, thus, by Eq. (33) and the reversibility of the chain 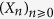 under 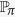, we have


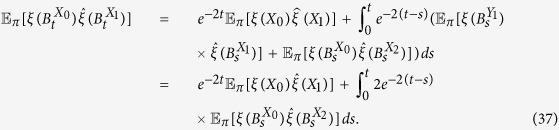


Integrating both sides of this equation with respect to *t* over (0, ∞) implies that


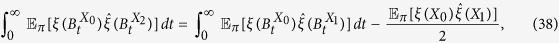


which, by Eqs (29), (34), and (35), gives Eq. (30).

The proof of the one remaining equation, Eq. (31), is similar except that we have to take into account the fact that 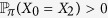 when applying Eq. (33). By reversibility, (*Y*_1_, *X*_0_, *X*_1_, *X*_2_) and (*X*_3_, *X*_2_, *X*_1_, *X*_0_) have the same distribution under 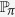. Therefore, by Eq. (33), it follows that


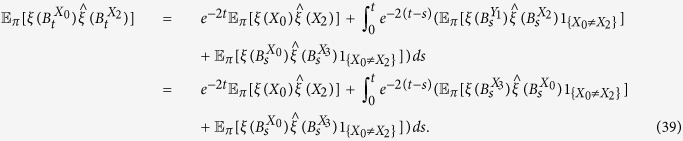


Integrating both sides of this equation with respect to *t* over (0, ∞) yields


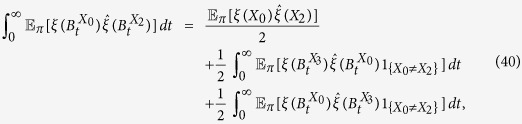


from which we obtain


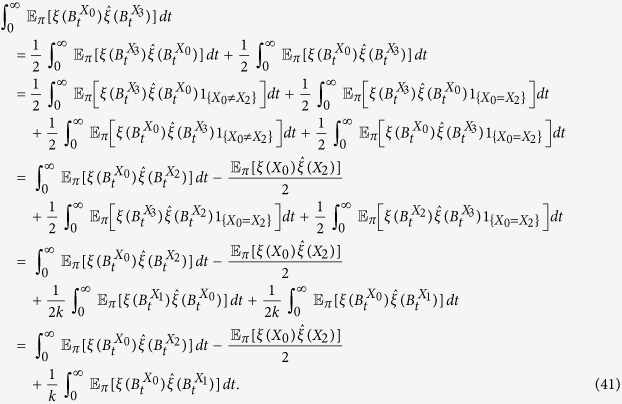


The first and last equalities follow from reversibility, the third equality from Eq. (40), and the fourth equality from the Markov property of 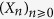 at *n* = 0, 2 and the fact that 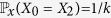 since the graph is regular. Eq. (31) then follows from Eqs (29), (30), (36) and (41). □

We are now in a position to prove the first of our main results:

**Theorem 3.** In the donation game, for any configuration, *ξ*, we have the following expansion as *w* → 0^+^:


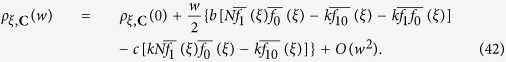


*Proof*. By Lemma 1, it suffices to obtain the coefficient of *w*, i.e. the first order term, on the right-hand side of Eq. (22). Since the game under consideration is the donation game, a simple calculation gives


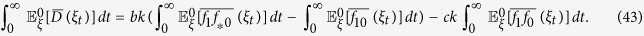


Therefore, Eq. (42) follows from the calculations of Lemma 2, which completes the proof. □

From Theorem 3, we see that, for small *w* > 0,


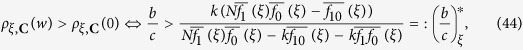


which gives the critical benefit-to-cost ratio of Eq. (7).

#### Structure coefficients

We now turn to a generalization of the critical benefit-to-cost ratio for arbitrary 2 × 2 games in which the payoff matrix is given by Eq. (12). Our main result is the following:

**Theorem 4.** 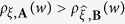 for all sufficiently small *w* > 0 if and only if


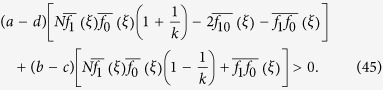


*Proof*. By the neutrality of the voter model, we have 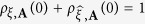, thus


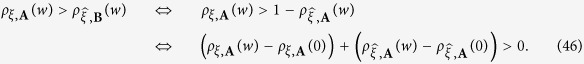


By the first-order expansion of Eq. (23), it follows that, for small *w* > 0,


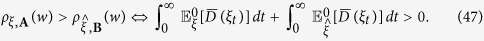


By Eqs (22) and (23) and the neutrality of the voter model, we have


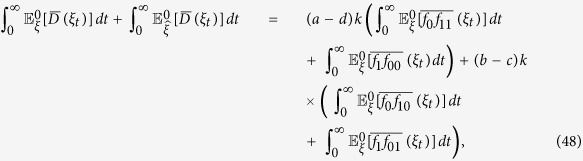


and all that remains is to determine the coefficients of *a* − *d* and *b* − *c* in Eq. (48). By considering Eq. (48) with the payoff values of the donation game rather than an arbitrary 2 × 2 game, we obtain


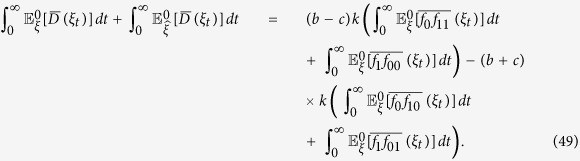


Thus, using Theorem 3, we see that, when *b* = −*c*,


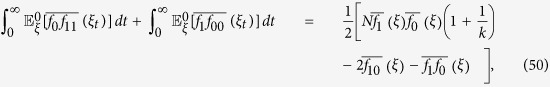


and, when *b* = *c*,


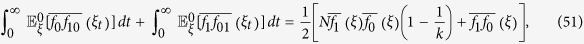


from which we obtain Eq. (45). □

In other words, 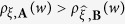 for all sufficiently small *w* > 0 if and only if


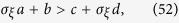


where *σ*_*ξ*_ is the structure coefficient given by


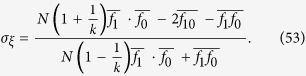


A simple calculation shows that


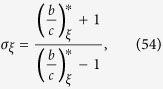


and, moreover, when the payoffs for the game are given by Eq. (13), this result reduces to Eq. (44).

### BD updating

Under BD updating, a player is first chosen to reproduce with probability proportional to fitness (effective payoff). A neighbor of the reproducing player is then chosen uniformly-at-random for death, and the offspring of the reproducing player fills this vacancy. The rate at which the player at vertex *x* is replaced by an *i*-player is then


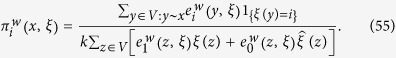


The neutral version of this process (*w* = 0) is a perturbation of the voter model, and we can use techniques similar to those used for DB updating to establish our main results for BD updating.

#### Critical benefit-to-cost ratios

Again, we first need a technical lemma:

**Lemma 5.** For *i, j, l* ∈ {0, 1}, *x* ∈ *V*, and *ξ* a configuration of cooperators and defectors, let


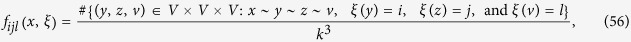


and form the averages 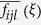 via Eq. (16). For any configuration, *ξ*, we have the following first-order expansion as *w* → 0^+^:


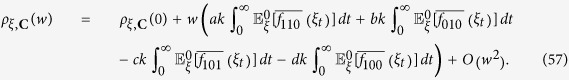


*Proof*. The first-order expansion of Eq. (23) is valid under BD updating as well [see ref. 39, Theorem 3.8], except that the function 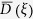 of Eqs (24)–(25) is defined in terms of the rates 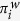 for BD updating rather than for DB updating. Writing 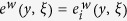 whenever *ξ*(*y*) = *i*, we find that


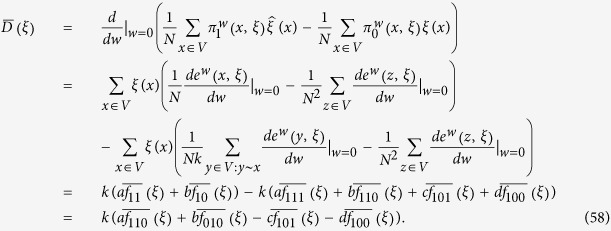


We then obtain Eq. (57) by applying this calculation to the first-order expansion of Eq. (23). □

Our main result for BD updating is the following:

**Theorem 6.** In the donation game, for any configuration, *ξ*, we have the following expansion as *w* → 0^+^:


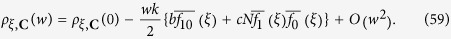


*Proof*. For the donation game, the function 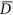 of Eq. (58) simplifies to


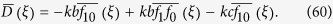


Therefore, by the calculations of Lemma 2, we see that


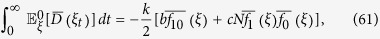


which gives Eq. (59) and completes the proof. □

Since 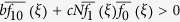 for each mixed state, *ξ*, it follows that *ρ*_*ξ*,**C**_(*w*) < *ρ*_*ξ*,**C**_(0) for all sufficiently small *w* > 0 whenever *ξ* is not an absorbing state, so cooperation is always suppressed by weak selection under BD updating.

#### Structure coefficients

Although cooperation is never favored by weak selection under BD updating, we can still write down a condition for selection to favor strategy *A* in an arbitrary 2 × 2 game whose payoff matrix is given by Eq. (12):

**Theorem 7.** 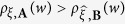 for all sufficiently small *w* > 0 if and only if


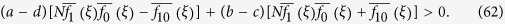


*Proof*. The same argument given in the proof of Theorem 4 shows that Eq. (62) is equivalent to


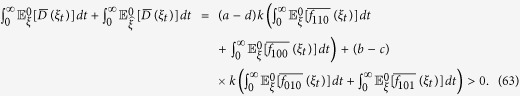


Solving for the coefficients of *a* − *d* and *b* − *c* as in the proof of Theorem 4 gives Eq. (62). □

Written differently, 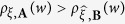 for all sufficiently small *w* > 0 if and only if


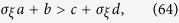


where *σ*_*ξ*_ is the structure coefficient given by


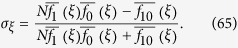


### Strategic placement of cooperators for DB updating

We turn now to the consequences of Theorem 3 for DB updating.

**Proposition 8.** Let *k* ≥ 2 be fixed. In the limit of large population size, *N* → ∞, the critical benefit-to-cost ratio converges uniformly to *k* over all *k*-regular graphs, *G*, of size *N* and all configurations, *ξ*, on *G*.

Proposition 8 follows immediately from the following technical result:

**Lemma 9.** For fixed *k* ≥ 2 and for *N* > 4*k*^2^ +1 such that there exists a *k*-regular graph with *N* vertices,


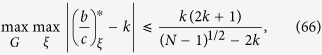


where *G* ranges over all *k*-regular graphs on *N* vertices, and, for each *G, ξ* ranges over all mixed configurations.

*Proof*. By the Cauchy-Schwarz inequality and the reversibility of the random walk, both 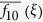 and 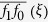 are bounded by 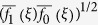. Therefore, for any such *G* and any mixed *ξ*, it follows from Eq. (7) that


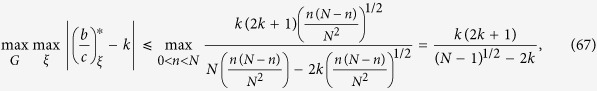


which completes the proof. □

**Proposition 10.** For all configurations obtained by placing an arbitrary (but fixed) number of cooperators uniformly at random, the critical benefit-to cost ratio is given by Eq. (2) in the main text.

*Proof*. Fix a *k*-regular graph with *N* vertices and, for 0 < *n* < *N*, let **u**_*n*_ denote the uniform distribution on the set of configurations, *ξ*, with exactly *n* cooperators. Since **u**_*n*_ is independent of the graph geometry,


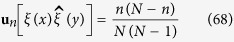


whenever *x* ≠ *y*. Therefore, by the definitions of *f*_*i*_ and *f*_*ij*_ in Eqs (14) and (15),


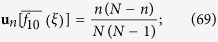



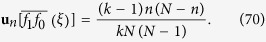


It follows from Eq. (7) in the main text that the critical benefit-to-cost ratio for **u**_*n*_ is


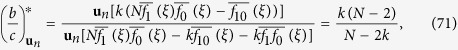


which is independent of *n* and coincides with Eq. (2). Furthermore, one can use Eqs (69) and (70) to see that the coefficient of *w* on the right-hand side of Eq. (42) under the random placement **u**_*n*_ is equal to


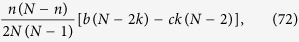


which is consistent with Theorem 1 in ref. 39. □

**Remark 2** (Neutrality of random configurations).On an arbitrary finite, connected social network, there is still an expansion in *w* for fixation probabilities that generalizes Eq. (23) [see ref. 39, Theorem 3.8]. Moreover, for the donation game and a configuration given randomly by **u**_*n*_, this expansion takes the form


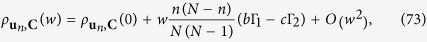


where Γ_1_ and Γ_2_ are constants that are independent of *n, b*, and *c*. (See the proof of Lemma 3.1 and the discussion of ‘Bernoulli transforms’ on p. 655–656 in ref. 39. For the linearity of the coefficient of *w* in *b* and *c*, see also ref. 34). By Eq. (73), the benefit-to-cost ratio for any *n*-random configuration is independent of *n*, so random configurations with more cooperators are neither more nor less conducive to cooperation than those with fewer.

For a fixed graph, *G*, let *N*_0_ be the maximum number of vertices that can be chosen in such a way that no two of these vertices are within two steps of one another. We say that a subset of vertices with this property is isolated. If the defectors in a configuration lie on isolated vertices, then we say that defectors are isolated.

**Proposition 11.** If *N* > 2*k*, then cooperation can be favored for a mixed configuration with *n* cooperators whenever either 1 ≤ *n* ≤*N*_0_ + 1 or 1 ≤ *N* − *n* ≤ *N*_0_ + 1.

*Proof*. For any configuration, *ξ*, with *n* cooperators, we have the inequalities


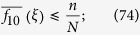



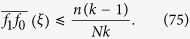


In Eqs (74) and (75), equality is obtained by a configuration with *n* isolated cooperators. Indeed, 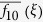 and 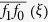 depend on the number of cooperator-defector paths and the number of cooperator-anything-defector paths in *ξ*, respectively, and each such path is defined by either an edge or two incident edges. On the other hand, at least one of these inequalities is strict whenever *ξ* does not have isolated cooperators: If two cooperators are adjacent to one another, then Eq. (74) is strict; if two cooperators are adjacent to the same defector, then Eq. (75) is strict. In order to establish the proposition, we need to show that


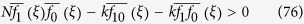


since, then, the critical benefit-to-cost ratio of Eq. (7) is finite. Moreover, since Eq. (76) is invariant under conjugation, it suffices to consider configurations with *n* cooperators, where 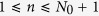. By Eqs (74)–(75), 129,


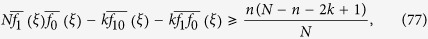


so it suffices to establish the inequality 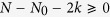.

Suppose, on the other hand, that *N* − *N*_0_ − 2*k* < 0. By the definition of *N*_0_, we can then find a configuration with *N* − 2*k* + 1 isolated cooperators. Since each of these cooperators has *k* neighboring defectors, and since none of these defectors have more than one cooperator as a neighbor, we have


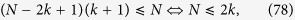


which contradicts the assumption that *N* > 2*k*, as desired. □

**Remark 3.** The proof of Proposition 11 shows that whenever *N* > 2*k*, in fact 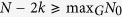 holds, where *G* ranges over all *k*-regular graphs on *N* vertices. This lower bound, max*GN*_0_, is sharp, which can be seen from the graph in Fig. 2(b) since this graph has size 9, is 4-regular, and satisfies *N*_0_ = 1.

**Proposition 12.** Suppose that *N* > 2*k*. Let *ξ* and *ξ*′ be configurations with *n* and *n* − 1 cooperators, respectively, such that defectors under both configurations are isolated. Then,


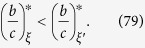


*Proof*. Since the defectors in both *ξ* and *ξ*′ are isolated, we have


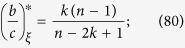



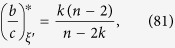


and it follows at once that 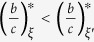 since *k* ≥ 2, as desired. □

As a consequence of Proposition 12, we see that among the configurations with an isolated strategy (cooperators or defectors), the minimum critical benefit-to-cost ratio is attained by any configuration with just a single cooperator.

**Proposition 13.** For a *k*-regular graph with *N* > 2*k*, we have the following:

- if *N*_0_ ≥ 2, then, for any *n* with 2 ≤ *n* ≤ *N*_0_, there exists a configuration with *n* cooperators whose critical benefit-to-cost ratio is smaller than that of a random configuration;
- for any configuration with exactly two cooperators, such that, furthermore, these two cooperators are neighbors, cooperation can be favored by weak selection. Moreover, the critical benefit-to-cost ratio for this configuration is smaller than that of a random configuration.

*Proof*. By Proposition 12, the critical benefit-to-cost ratio for any configuration with *n* ≥ 2 isolated cooperators is greater than the critical benefit-to-cost ratio for any configuration with just a single cooperator. Recall now that this ratio for one cooperator is the same as the ratio for *n* randomly-placed cooperators^39^. By Eq. (43), the critical benefit-to-cost ratio for *ξ* is of the form 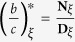 for some voter-model expectations, **N**_*ξ*_ and **D**_*ξ*_. Therefore, by a simple averaging argument, we see that for each *n* with 2 ≤ *n* ≤ *N*_0_, there must exist a configuration with *n* cooperators whose critical ratio is smaller than that of a random configuration, Eq. (2), which completes the proof of part 1 of the proposition.

Let *ξ* be a configuration with two cooperators placed at adjacent vertices, *x* and *y*. If *T*(*x, y*) is the number of vertices adjacent to both *x* and *y*, then straightforward calculations give


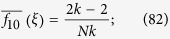



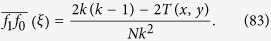


It then follows from the definition of the critical benefit-to-cost ratio that


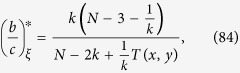


which is smaller than the ratio for random placement, Eq. (2), because *N* > 2*k*, which gives part 2. □

### Examples

**Figure 4:**
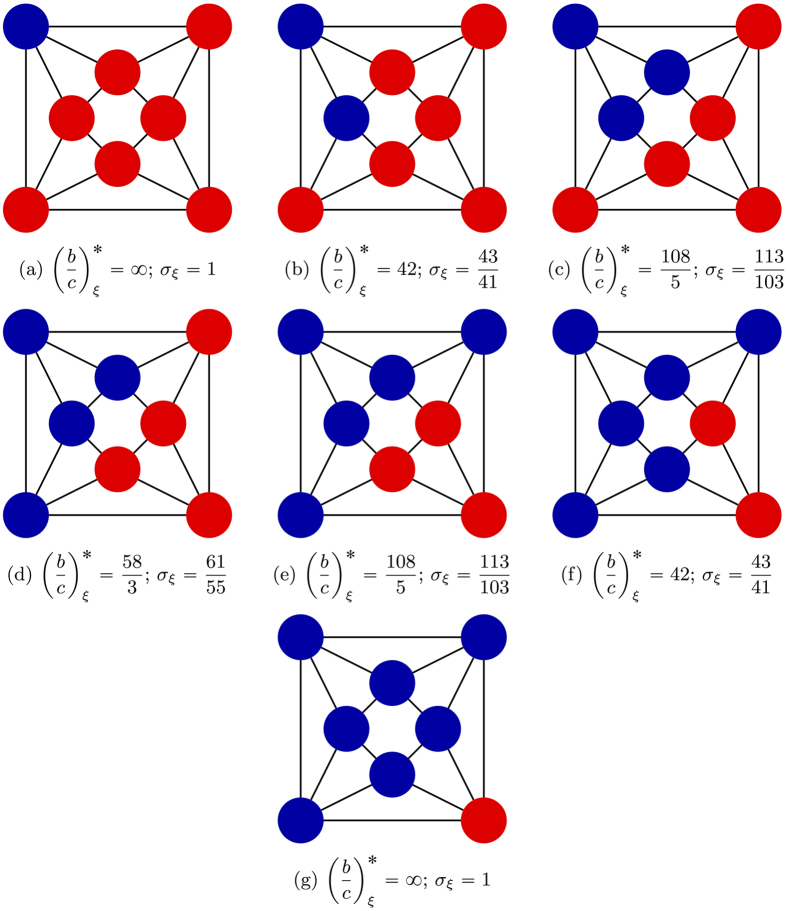
Cooperator-defector configurations on a 4-regular graph with 8 vertices and diameter 2. Starting from one cooperator in (**a**), a single cooperator is added in each subsequent panel. Although cooperation can never be favored by selection when starting from a state with a single mutant (**a**) or a single defector (**g**), it can be favored in the other states, (**b**–**f**), since the critical benefit-to-cost ratios are all finite in those panels.

**Figure 5:**
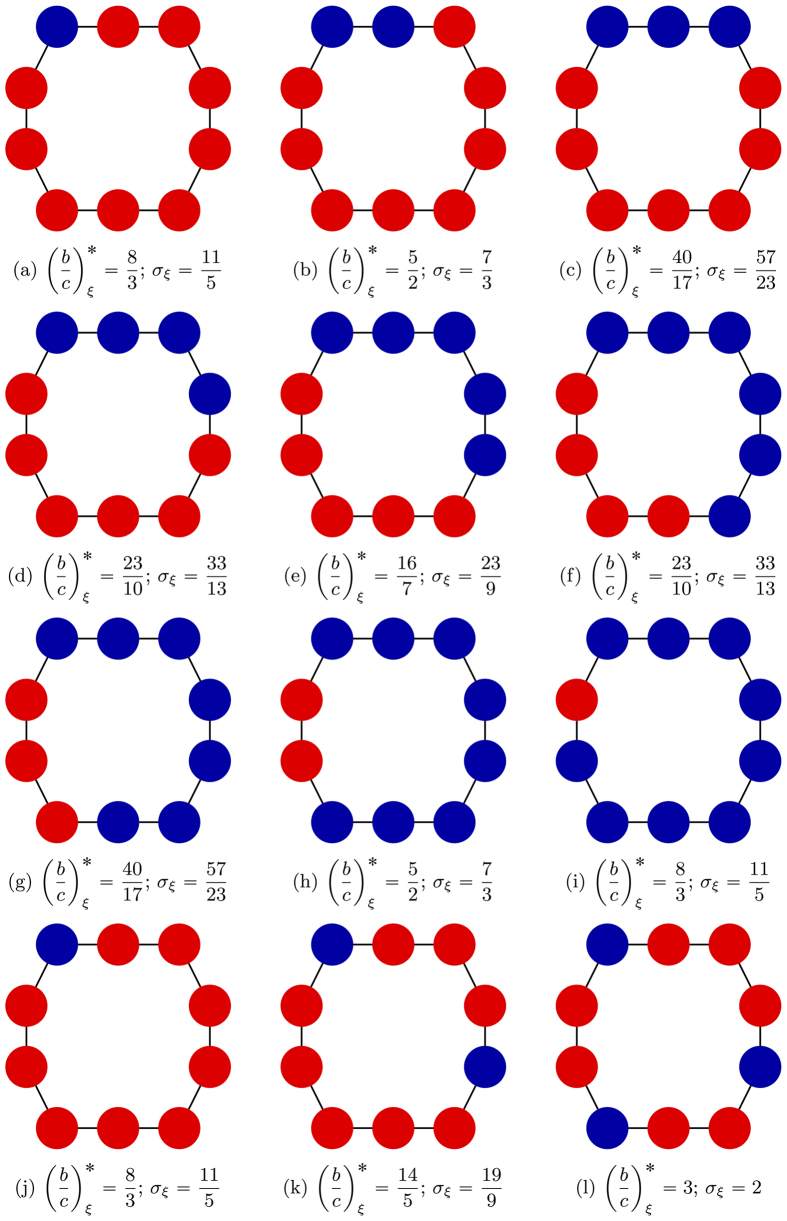
The effects of adding cooperators to the initial condition on a cycle with 10 vertices. In panels (a–i), cooperators are added sequentially, with each new cooperator neighboring a cooperator in the previous configuration. These panels clearly demonstrate that a configuration and its conjugate have the same critical ratio and structure coefficient. Panels (j–l) show that when cooperators are added in a different order, the critical ratios can increase rather than decrease. The configurations of (j–l) each have isolated cooperators.

In Figs 4 and 5, we give examples of the relationship between the configuration and the critical benefit-to-cost ratio on three small graphs.

## Additional Information

**How to cite this article**: Chen, Y.-T. *et al*. Fixation Probabilities for Any Configuration of Two Strategies on Regular Graphs. *Sci. Rep.* **6**, 39181; doi: 10.1038/srep39181 (2016).

**Publisher's note:** Springer Nature remains neutral with regard to jurisdictional claims in published maps and institutional affiliations.

## References

[b1] Brosnan S. F. & Bshary R. Cooperation and deception: from evolution to mechanisms. Philos. Trans. R. Soc. London, Ser. B 365, 2593–2598 (2010). 20679104 10.1098/rstb.2010.0155 PMC2942876

[b2] Nowak M. A. Five rules for the evolution of cooperation. Science 314, 1560–1563 (2006b). 17158317 10.1126/science.1133755 PMC3279745

[b3] Zaggl M. A. Eleven mechanisms for the evolution of cooperation. J. Inst. Econ. 10, 197–230 (2013).

[b4] Nowak M. A. Evolutionary Dynamics: Exploring the Equations of Life (Belknap Press, 2006a).

[b5] Nowak M. A., Sasaki A., Taylor C. & Fudenberg D. Emergence of cooperation and evolutionary stability in finite populations. Nature 428, 646–650 (2004). 15071593 10.1038/nature02414

[b6] Brauchli K., Killingback T. & Doebeli M. Evolution of cooperation in spatially structured populations. J. Theor. Biol. 200, 405–417 (1999). 10525399 10.1006/jtbi.1999.1000

[b7] Durrett R. Probability Models for DNA Sequence Evolution (Springer: New York, 2002).

[b8] Fu F., Nowak M. & Hauert C. Invasion and expansion of cooperators in lattice populations: prisoner’s dilemma vs. snowdrift games. J. Theor. Biol. 266, 358–366 (2010). 20619271 10.1016/j.jtbi.2010.06.042 PMC2927800

[b9] Helbing D., Szolnoki A., Perc M. & Szabó G. Punish, but not too hard: how costly punishment spreads in the spatial public goods game. New J. Phys. 12, 083005 (2010).

[b10] Hutson V. & Vickers G. T. Backward and forward traveling waves in evolutionary games. Methods Appl. Anal. 9, 159–176 (2002).

[b11] Hwang S.-H., Katsoulakis M. & Rey-Bellet L. Deterministic equations for stochastic spatial evolutionary games. Theor. Econ. 8, 829–874 (2013).

[b12] Ifti M., Killingback T. & Doebeli M. Effects of neighbourhood size and connectivity on the spatial Continuous Prisoner’s Dilemma. J. Theor. Biol. 231, 97–106 (2004). 15363932 10.1016/j.jtbi.2004.06.003

[b13] Jansen V. A. A. & van Baalen M. Altruism through beard chromodynamics. Nature 440, 663–666 (2006). 16572169 10.1038/nature04387

[b14] Kaveh K., Komarova N. L. & Kohandel M. The duality of spatial death-birth and birth-death processes and limitations of the isothermal theorem. R. Soc. Open Sci. 2, 140465 (2015). 26064637 10.1098/rsos.140465 PMC4448870

[b15] Killingback T. & Doebeli M. Spatial evolutionary game theory: hawks and doves revisited. Proc. R. Soc. B 263, 1135–1144 (1996).

[b16] Killingback T. & Doebeli M. Self-organized criticality in spatial evolutionary game theory. J. Theor. Biol. 191, 335–340 (1998). 9631572 10.1006/jtbi.1997.0602

[b17] Komarova N. L. Spatial stochastic models for cancer initiation and progression. Bull. Math. Biol. 68, 1573–1599 (2006). 16832734 10.1007/s11538-005-9046-8

[b18] Lindgren K. & Nordahl M. G. Evolutionary dynamics of spatial games. Physica D 75, 292–309 (1994).

[b19] Nowak M. A. & May R. M. Evolutionary games and spatial chaos. Nature 359, 826–829 (1992).

[b20] Nowak M. A., Tarnita C. E. & Antal T. Evolutionary dynamics in structured populations. Philos. Trans. R. Soc. London, Ser. B 365, 19–30 (2009). 10.1098/rstb.2009.0215 PMC2842709 20008382

[b21] Perc M. & Szolnoki A. Social diversity and promotion of cooperation in the spatial prisoner’s dilemma game. Phys. Rev. E 77 (2008). 10.1103/PhysRevE.77.011904 18351873

[b22] Ranta E., Lundberg P. & Kaitala V. Spatial games in Ecology of Populations 267–299 (Cambridge University Press, 2005).

[b23] Roca C. P., Cuesta J. A. & Sánchez A. Evolutionary game theory: temporal and spatial effects beyond replicator dynamics. Phys. Life Rev. 6, 208–249 (2009). 20416850 10.1016/j.plrev.2009.08.001

[b24] Schreiber S. J. & Killingback T. P. Spatial heterogeneity promotes coexistence of rock–paper–scissors metacommunities. Theor. Pop. Biol. 86, 1–11 (2013). 23474219 10.1016/j.tpb.2013.02.004

[b25] Szabó G., Antal T., Szabó P. & Droz M. Spatial evolutionary prisoner’s dilemma game with three strategies and external constraints. Phys. Rev. E 62, 1095–1103 (2000). 10.1103/physreve.62.1095 11088565

[b26] Szabó G. & Hauert C. Phase transitions and volunteering in spatial public goods games. Phys. Rev. Lett. 89 (2002). 10.1103/PhysRevLett.89.118101 12225171

[b27] Szolnoki A. & Perc M. Reward and cooperation in the spatial public goods game. EPL 92, 38003 (2010). 10.1103/PhysRevE.92.012819 26274237

[b28] Van Cleve J. & Lehmann L. Stochastic stability and the evolution of coordination in spatially structured populations. Theor. Pop. Biol. 89, 75–87 (2013). 23999503 10.1016/j.tpb.2013.08.006

[b29] Rand D. G., Nowak M. A., Fowler J. H. & Christakis N. A. Static network structure can stabilize human cooperation. Proc. Natl. Acad. Sci. USA 111, 17093–17098 (2014). 25404308 10.1073/pnas.1400406111 PMC4260616

[b30] Débarre F., Hauert C. & Doebeli M. Social evolution in structured populations. Nat. Commun. 5 (2014). 10.1038/ncomms4409 24598979

[b31] Allen B. & Nowak M. A. Games on graphs. EMS Surv. Math. Sci. 1, 113–151 (2014).

[b32] Ohtsuki H., Hauert C., Lieberman E. & Nowak M. A. A simple rule for the evolution of cooperation on graphs and social networks. Nature 441, 502–505 (2006). 16724065 10.1038/nature04605 PMC2430087

[b33] Ohtsuki H. & Nowak M. A. The replicator equation on graphs. J. Theor. Biol. 243, 86–97 (2006). 16860343 10.1016/j.jtbi.2006.06.004 PMC2430083

[b34] Tarnita C. E., Ohtsuki H., Antal T., Fu F. & Nowak M. A. Strategy selection in structured populations. J. Theor. Biol. 259, 570–581 (2009b). 19358858 10.1016/j.jtbi.2009.03.035 PMC2710410

[b35] Sigmund K. The Calculus of Selfishness (Princeton University Press, 2010).

[b36] Maynard Smith J. Evolution and the Theory of Games (Cambridge University Press, 1982).

[b37] Broom M. & Rychtář J. A general framework for analysing multiplayer games in networks using territorial interactions as a case study. J. Theor. Biol. 302, 70–80 (2012). 22406262 10.1016/j.jtbi.2012.02.025

[b38] Broom M., Rychtář J. & Stadler B. T. Evolutionary dynamics on graphs - the effect of graph structure and initial placement on mutant spread. J. Stat. Theory Pract. 5, 369–381 (2011).

[b39] Chen Y.-T. Sharp benefit-to-cost rules for the evolution of cooperation on regular graphs. Ann. Appl. Probab. 23, 637–664 (2013).

[b40] Lieberman E., Hauert C. & Nowak M. A. Evolutionary dynamics on graphs. Nature 433, 312–316 (2005). 15662424 10.1038/nature03204

[b41] Maciejewski W., Fu F. & Hauert C. Evolutionary game dynamics in populations with heterogenous structures. PLoS Comp. Biol. 10, e1003567 (2014). 10.1371/journal.pcbi.1003567 PMC3998889 24762474

[b42] Santos F. C., Santos M. D. & Pacheco J. M. Social diversity promotes the emergence of cooperation in public goods games. Nature 454, 213–216 (2008). 18615084 10.1038/nature06940

[b43] Shakarian P., Roos P. & Johnson A. A review of evolutionary graph theory with applications to game theory. Biosystems 107, 66–80 (2012) 22020107 10.1016/j.biosystems.2011.09.006

[b44] Szabó G. & Fáth G. Evolutionary games on graphs. Phys. Rep. 446, 97–216 (2007).

[b45] Szolnoki A., Perc M. & Szabó G. Topology-independent impact of noise on cooperation in spatial public goods games. Phys. Rev. E 80 (2009). 10.1103/PhysRevE.80.056109 20365045

[b46] Taylor P. D., Day T. & Wild G. Evolution of cooperation in a finite homogeneous graph. Nature 447, 469–472 (2007). 17522682 10.1038/nature05784

[b47] van Veelen M., Garcia J., Rand D. G. & Nowak M. A. Direct reciprocity in structured populations. Proc. Natl. Acad. Sci. USA 109, 9929–9934 (2012). 22665767 10.1073/pnas.1206694109 PMC3382515

[b48] Ohtsuki H., Nowak M. A. & Pacheco J. M. Breaking the symmetry between interaction and replacement in evolutionary dynamics on graphs. Phys. Rev. Lett. 98 (2007a). 10.1103/PhysRevLett.98.108106 PMC2387227 17358573

[b49] Ohtsuki H., Pacheco J. M. & Nowak M. A. Evolutionary graph theory: Breaking the symmetry between interaction and replacement. J. Theor. Biol. 246, 681–694 (2007b). 17350049 10.1016/j.jtbi.2007.01.024 PMC2396517

[b50] Pacheco J. M., Pinheiro F. L. & Santos F. C. Population structure induces a symmetry breaking favoring the emergence of cooperation. PLoS Comp. Biol. 5, e1000596 (2009). 10.1371/journal.pcbi.1000596 PMC2782104 20011116

[b51] Antal T., Ohtsuki H., Wakeley J., Taylor P. D. & Nowak M. A. Evolution of cooperation by phenotypic similarity. Proc. Natl. Acad. Sci. USA 106, 8597–8600 (2009). 19416902 10.1073/pnas.0902528106 PMC2688992

[b52] Tarnita C. E., Antal T., Ohtsuki H. & Nowak M. A. Evolutionary dynamics in set structured populations. Proc. Natl. Acad. Sci. USA 106, 8601–8604 (2009a). 19433793 10.1073/pnas.0903019106 PMC2689033

[b53] Wardil L. & Hauert C. Origin and structure of dynamic cooperative networks. Sci. Rep. 4 (2014). 10.1038/srep05725 PMC4101522 25030202

[b54] Wu B. . Evolution of cooperation on stochastic dynamical networks. PLoS ONE 5, e11187 (2010b). 20614025 10.1371/journal.pone.0011187 PMC2894855

[b55] Moran P. A. P. Random processes in genetics. Math. Proc. Cambridge Philos. Soc. 54, 60–71 (1958).

[b56] Akashi H., Osada N. & Ohta T. Weak selection and protein evolution. Genetics 192, 15–31 (2012). 22964835 10.1534/genetics.112.140178 PMC3430532

[b57] Fu F., Wang L., Nowak M. A. & Hauert C. Evolutionary dynamics on graphs: efficient method for weak selection. Phys. Rev. E 79 (2009). 10.1103/PhysRevE.79.046707 PMC2735202 19518380

[b58] Mullon C. & Lehmann L. The robustness of the weak selection approximation for the evolution of altruism against strong selection. J. Evol. Biol. 27, 2272–2282 (2014). 25146161 10.1111/jeb.12462

[b59] Wild G. & Traulsen A. The different limits of weak selection and the evolutionary dynamics of finite populations. J. Theor. Biol. 247, 382–390 (2007). 17462673 10.1016/j.jtbi.2007.03.015

[b60] Wu B., Altrock P. M., Wang L. & Traulsen A. Universality of weak selection. Phys. Rev. E 82 (2010a). 10.1103/PhysRevE.82.046106 21230344

[b61] Wu B., García J., Hauert C. & Traulsen A. Extrapolating weak selection in evolutionary games. PLoS Comp. Biol. 9, e1003381 (2013). 10.1371/journal.pcbi.1003381 PMC3854678 24339769

[b62] Fudenberg D. & Imhof L. A. Imitation processes with small mutations. J. Econ. Theory 131, 251–262 (2006).

[b63] Wu B., Gokhale C. S., Wang L. & Traulsen A. How small are small mutation rates? J. Math. Biol. 64, 803–827 (2011). 21626364 10.1007/s00285-011-0430-8

[b64] Bromham L. Why do species vary in their rate of molecular evolution? Biol. Lett. 5, 401–404 (2009). 19364710 10.1098/rsbl.2009.0136 PMC2679939

[b65] Loewe L. & Hill W. G. The population genetics of mutations: good, bad and indifferent. Philos. Trans. R. Soc. London, Ser. B 365, 1153–1167 (2010). 20308090 10.1098/rstb.2009.0317 PMC2871823

[b66] Lynch M. Evolution of the mutation rate. Trends Genet. 26, 345–352 (2010). 20594608 10.1016/j.tig.2010.05.003 PMC2910838

[b67] Otto S. P. & Hastings I. M. Mutation and selection within the individual in Mutation and Evolution 507–524 (Springer, 1998). 9766963

[b68] Hauert C. & Imhof L. Evolutionary games in deme structured, finite populations. J. Theor. Biol. 299, 106–112 (2012). 21704639 10.1016/j.jtbi.2011.06.010

[b69] Miękisz J. Evolutionary game theory and population dynamics in Lecture Notes in Mathematics 269–316 (Springer, 2008) (2008).

[b70] Ohtsuki H. Evolutionary games in Wright’s island model: kin selection meets evolutionary game theory. Evolution 64, 3344–3353 (2010). 20812974 10.1111/j.1558-5646.2010.01117.x

[b71] Pichugin Y., Gokhale C. S., Garcia J., Traulsen A. & Rainey P. B. Modes of migration and multilevel selection in evolutionary multiplayer games. J. Theor. Biol. 387, 144–153 (2015). 26456203 10.1016/j.jtbi.2015.09.027

[b72] Nagao M., Sugimura T. & Matsushima T. Environmental mutagens and carcinogens. Annu. Rev. Genet. 12, 117–159 (1978). 371523 10.1146/annurev.ge.12.120178.001001

[b73] Nunney L. The real war on cancer: the evolutionary dynamics of cancer suppression. Evol. Appl. 6, 11–19 (2012). 23396311 10.1111/eva.12018 PMC3567467

[b74] McAvoy A. & Hauert C. Structural symmetry in evolutionary games. J. R. Soc. Interface 12, 20150420 (2015). 26423436 10.1098/rsif.2015.0420 PMC4614485

[b75] Hindersin L., Möller M., Traulsen A. & Bauer B. Exact numerical calculation of fixation probability and time on graphs. Biosystems 150, 87–91 (2016). 27555086 10.1016/j.biosystems.2016.08.010

[b76] Ibsen-Jensen R., Chatterjee K. & Nowak M. A. Computational complexity of ecological and evolutionary spatial dynamics. Proc. Natl. Acad. Sci. USA 112, 15636–15641 (2015). 26644569 10.1073/pnas.1511366112 PMC4697423

[b77] Voorhees B. Birth-death fixation probabilities for structured populations. Proc. R. Soc. A 469, 20120248 (2013).

[b78] Grafen A. An inclusive fitness analysis of altruism on a cyclical network. J. Evol. Biol. 20, 2278–2283 (2007). 17956390 10.1111/j.1420-9101.2007.01413.x

[b79] Grafen A. & Archetti M. Natural selection of altruism in inelastic viscous homogeneous populations. J. Theor. Biol. 252, 694–710 (2008). 18371985 10.1016/j.jtbi.2008.01.021

[b80] Liggett T. M. Interacting Particle Systems (Springer, 1985).

[b81] Rousset F. A minimal derivation of convergence stability measures. J. Theor. Biol. 221, 665–668 (2003). 12713948 10.1006/jtbi.2003.3210

[b82] Lessard S. & Ladret V. The probability of fixation of a single mutant in an exchangeable selection model. J. Math. Biol. 54, 721–744 (2007). 17252282 10.1007/s00285-007-0069-7

[b83] Ladret V. & Lessard S. Evolutionary game dynamics in a finite asymmetric two-deme population and emergence of cooperation. J. Theor. Biol. 255, 137–151 (2008). 18694762 10.1016/j.jtbi.2008.07.025

[b84] Chen Y.-T., Choi J. & Cox J. T. On the convergence of densities of finite voter models to the Wright–Fisher diffusion. Ann. Inst. Henri Poincaré Probab. Stat. 52, 286–322 (2016).

[b85] Frucht R. Graphs of degree three with a given abstract group. Can. J. Math. 1, 365–378 (1949).

